# Maxillary nerve compression in cynomolgus monkey *Macaca fascicularis*: altered somatic sensation and peripheral nerve firing

**DOI:** 10.1186/1471-2202-13-150

**Published:** 2012-12-13

**Authors:** Ning Guo, Xiyao Gu, Jun Zhao, Guoping Zhao, Meilei Jin, Hong Zou, Yuqiu Zhang, Zhiqi Zhao, Gang Jason Jin, Lei Yu

**Affiliations:** 1 Shanghai University of Traditional Chinese Medicine, and ShanghaiBio Corporation, Shanghai, China; 2 Institute of Neurobiology, Institutes of Brain Science and State Key Laboratory of Medical Neurobiology, Fudan University, Shanghai, 200032, China; 3 Shanghai Institutes for Biological Sciences, Chinese Academy of Sciences, Shanghai, China; 4 Department of Genetics & Center of Alcohol Studies, Rutgers University, 607 Allison Road, Piscataway, New Jersey, 08854, USA

**Keywords:** Maxillary nerve, Trigeminal nerve, Compression, Multiple-unit recording, Non-human primates

## Abstract

**Background:**

Trigeminal nerve is a major source of the sensory input of the face, and trigeminal neuropathology models have been reported in rodents with injury to branches of the maxillary or mandibular division of the trigeminal nerve. Non-human primates are neuroanatomically more closely related to human than rodents; however, nerve injury studies in non-human primates are limited.

**Results:**

We describe here a nerve injury model of maxillary nerve compression (MNC) in the cynomolgus macaque monkey, *Macaca fascicularis*, and the initial characterization of the consequences of damage to this trigeminal nerve branch. The nerve injury from the compression appeared to be mild, as we did not observe overt changes in home-cage behavior in the monkeys. When mechanical stimulation was applied to the facial area, monkeys with MNC displayed increased mechanical sensitivity, as the avoidance response scores were lower than those from the control animals. Such a change in mechanical sensitivity appeared to be somewhat bilateral, as the contralateral side also showed increased mechanical sensitivity, although the change on the ipsilateral side was more robust. Multiple-unit recording of the maxillary nerve showed a general pattern of increasing responsiveness to escalating force in mechanical stimulation on the contralateral side. Ipsilateral side of the maxillary nerve showed a lack of responsiveness to escalating force in mechanical stimulation, possibly reflecting a maximum stimulation threshold effect from sensitized nerve due to MNC injury.

**Conclusions:**

These results suggest that MNC may produce increased sensitivity of the ipsilateral maxillary nerve, and that this model may serve as a non-human primate model to evaluate the effect of injury to trigeminal nerve branches.

## Background

As a cranial nerve, the trigeminal nerve is a major source of the sensory input of the face, and pathological conditions in the trigeminal nerve often result in dire consequences such as trigeminal neuralgia. To better characterize such pathological conditions, animal models are valuable in aiding investigative studies. Trigeminal neuropathology models have been established, primarily in rodents via injury to infraorbital nerve, a branch of the maxillary division of the trigeminal nerve, and to mental nerve, a branch of the mandibular division of the trigeminal nerve. Behavioral changes reported using these models include increased facial grooming in rats receiving unilateral chronic constriction injury (CCI) of the infraorbital nerve [1] and bilateral CCI of the mental nerve [2]. In mice with partial infraorbital nerve ligation injury, a temporary increase in grooming was observed [3]. An initial increase of mechanical threshold early after CCI surgery of the infraorbital nerve followed by a dramatic decrease of mechanical threshold in rats was observed on ipsilateral side of the face [1,4]. Tight ligation of the mental nerve in mice induced an increase in mechanical threshold by post-operation day 2–3, and the threshold returned to normal by post-operative day 14 [5]. In rats with CCI of the infraorbital nerve, although the surgery was done on one side, the decrease in mechanical threshold was sometimes observed bilaterally with the ipsilateral side showing stronger effect than the contralateral side [1,6-8]. Similar results were obtained by photochemical reaction induced partial ischemic injury to the infraorbital nerve in rats [9]. In two studies with the CCI injury of the infraorbital nerve, both sides showed a comparable decrease in mechanical threshold [10,11]. It has also been reported that only the ipsilateral side showed decrease in mechanical threshold with the contralateral side remaining normal [4]. Thermal hyperalgesia was observed on both sides of the face in the CCI model of the infraorbital nerve in rats, with the ipsilateral side demonstrating stronger responses to stimulus [12].

Non-human primates are neuroanatomically more closely related to human than rodents. However, nerve injury studies in non-human primates are limited. L7 spinal nerve ligation model was developed in a macaque monkey, *Macaca fascicularis*. The animals showed bilateral decrease in mechanical threshold of their feet with ipsilateral side showing stronger sensitization [13,14]. There have been only limited studies in non-human primates for injury models of trigeminal nerve branches, with one report of histological effects of trigeminal nerve radiosurgery in baboons [15].

In the present study, we used *Macaca fascicularis*, commonly known as the crab-eating macaque, long-tailed macaque, or cynomolgus monkey. We established an injury model of maxillary nerve compression (MNC), and report initial characterization of the behavioral and electrophysiological effect of MNC.

## Results

### Maxillary nerve compression as a model of nerve injury

**Figure 1:**
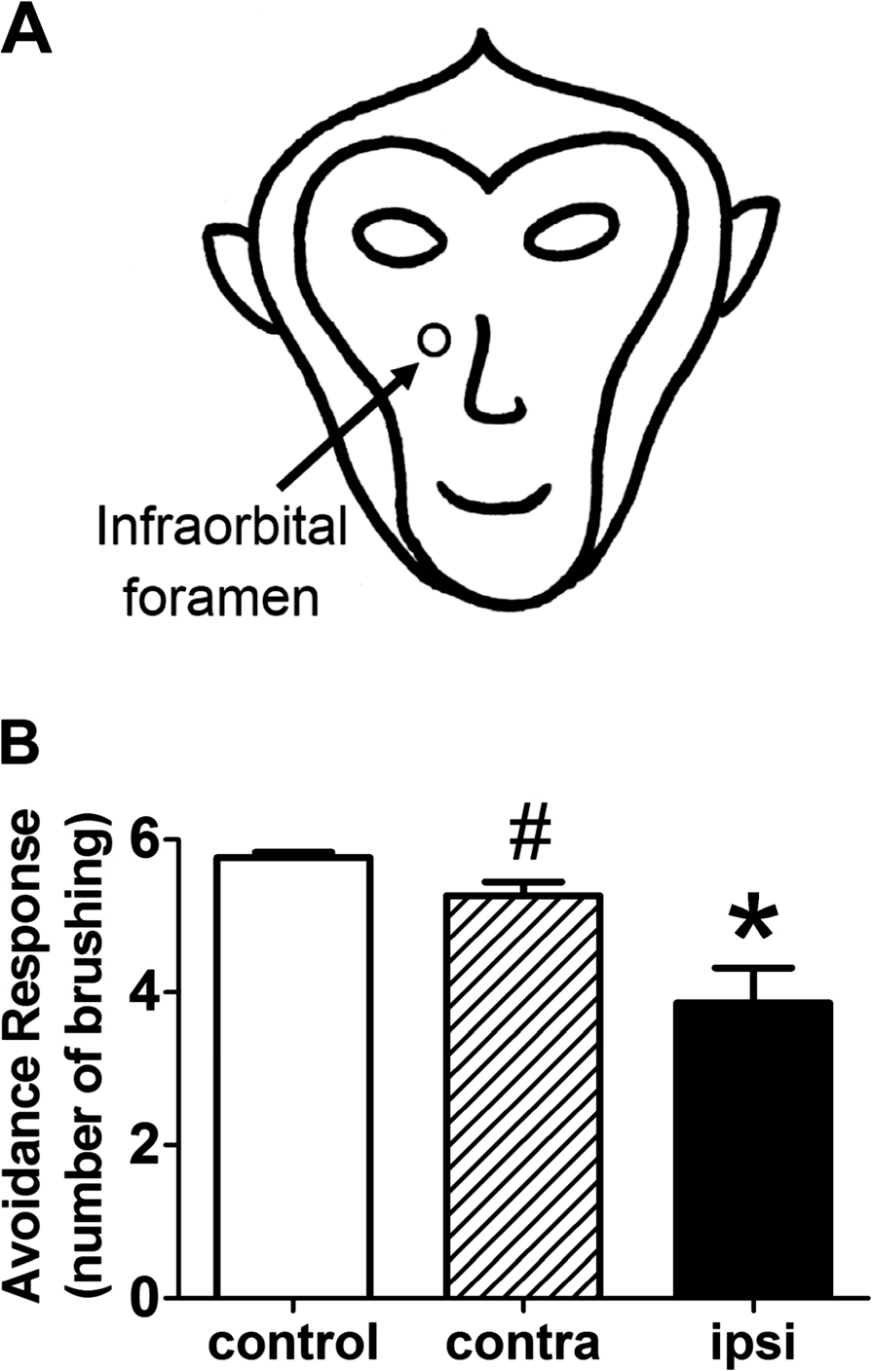
**Maxillary nerve compression in cynomolgus monkey and altered somatic sensation.** (**A**) Diagrammatic drawing of the face of *Macaca fascicularis*. The circle below the animal’s eye marks the position of infraorbital foramen, where the maxillary division of the trigeminal nerve penetrates the skull; the external portion of this maxillary nerve’s terminal branch is commonly referred to as the infraorbital nerve. (**B**) Sensitivity to facial mechanical stimulation in monkeys. Avoidance response in monkeys is shown as the number of brushing to the side of the face before a monkey turned its face away from that side to avoid further brushing stimulation. Responses are shown as mean ± SEM. Open bar, ‘control’ (control animals’ both left and right sides, n = 10); striped bar, ‘contra’ (contralateral side of the MNC animals, n = 7); filled bar, ‘ipsi’ (ipsilateral side of the MNC animals, n = 7). #, significantly different from control (p < 0.05, unpaired *t* test with Welch's correction). *, significantly different from both control and contralateral side (p < 0.05, unpaired *t* test with Welch's correction).

We generated a nerve injury model of maxillary nerve compression (MNC), using the non-human primate crab-eating macaque (*Macaca fascicularis*). As shown in Figure 1A, maxillary nerve, the maxillary division of the trigeminal nerve, penetrates the skull at the infraorbital foramen. An L-shaped stainless steel bar about 3 mm in length was made from a sterile 17-gauge injection needle by separating the metal shaft from the plastic syringe housing. After bending the metal shaft to approximate 90° angle, it was inserted into the infraorbital foramen, thus generating moderate compression upon the maxillary nerve.

The compression model did not appear to cause undue distress in the animals, as the monkeys did not show observable behavior changes, nor did they display signs of local inflammation or discomfort in the facial area after surgery. Prior to electrophysiological recording of the nerves, visual inspection of the general appearance of the maxillary nerve did not show noticeable difference between the ipsilateral vs. the contralateral side of the maxillary nerves; thus, the MNC was considered a mild nerve injury. Nerve histology was not performed, due to the fact that the maxillary nerves were used in electrophysiological recording, which rendered the nerve unsuitable for subsequent histological analysis.

### Effect of MNC on mechanical sensitivity of the facial area

To determine the effect of maxillary nerve compression, we tested monkey’s behavior response to mechanical stimulation. Gentle brushing generated with a soft-hair tooth-brush was applied to each side of the face, at the area adjacent to the upper lip where maxillary nerve innervates. If the monkey turned its head away from the side of brushing, it was considered a positive avoidance response, and the number of brushings eliciting the avoidance response was recorded as the avoidance response score for that side of the face. A cut-off maximum of six brushings was used, and the lack of response was recorded as a score of six. A total of eight rounds of brushing were performed for each monkey, with the order of left vs. right side randomized. The average of the eight scores was used as the avoidance response score for the side of the face.

In control animals, both sides of the face gave similar sensitivity, and there was no statistical difference between the avoidance response scores. Therefore, the response results were combined as ‘control’ (5.76 ± 0.07, mean ± SEM, n = 10), as shown in Figure 1B. In MNC animals, the ipsilateral side (the side of the face with MNC surgery) and the contralateral side showed avoidance response scores of 3.86 ± 0.46 (mean ± SEM, n = 7) and 5.26 ± 0.18 (mean ± SEM, n = 7), respectively. The avoidance response scores of the ipsilateral side were significantly different from those of both the contralateral side and the control (p < 0.05, unpaired *t* test with Welch's correction), while the avoidance response scores of the contralateral side were significantly different from those of the control (p < 0.05, unpaired *t* test with Welch's correction). These data indicate that unilateral MNC surgery resulted in bilateral reduction in avoidance response scores compared to those of the control animals, and that the ipsilateral side displayed a more robust reduction than the contralateral side. This suggests an increase in sensitivity to mechanical stimulation due to MNC.

### Effect of MNC on electrophysiological properties of the maxillary nerve

To examine the effect of MNC on the maxillary nerve itself, electrophysiological properties of the nerve was measured by multiple-unit recording, on a nerve bundle separated from the bulk of the maxillary nerve. To determine the receptive field of the nerve fibers being recorded, various spots in the upper lip and adjacent area were stimulated with a soft paint brush, and the spot that produced the strongest electric signal was considered representative of the receptive field of the nerve fibers being recorded. The positions of the receptive field on the contralateral (control) side vs. the ipsilateral (injured) side appeared to be symmetrical.

We first recorded the spontaneous activity of the nerve fibers. Thereafter, electrical response to mechanical stimulation applied to the receptive field was recorded. Successively stronger stimulation was achieved with a set of von Frey filaments, ranging in nominal force from 1g to 60g. We attempted to record nerve activities from both sides of the face, by simultaneous recording of both maxillary nerve bundles of an animal, and feeding the signals into separate channels of a DVD recorder. In two animals, we were successful in bilateral recording. In another two monkeys, we were only able to record the contralateral side of the maxillary nerve.

**Figure 2:**
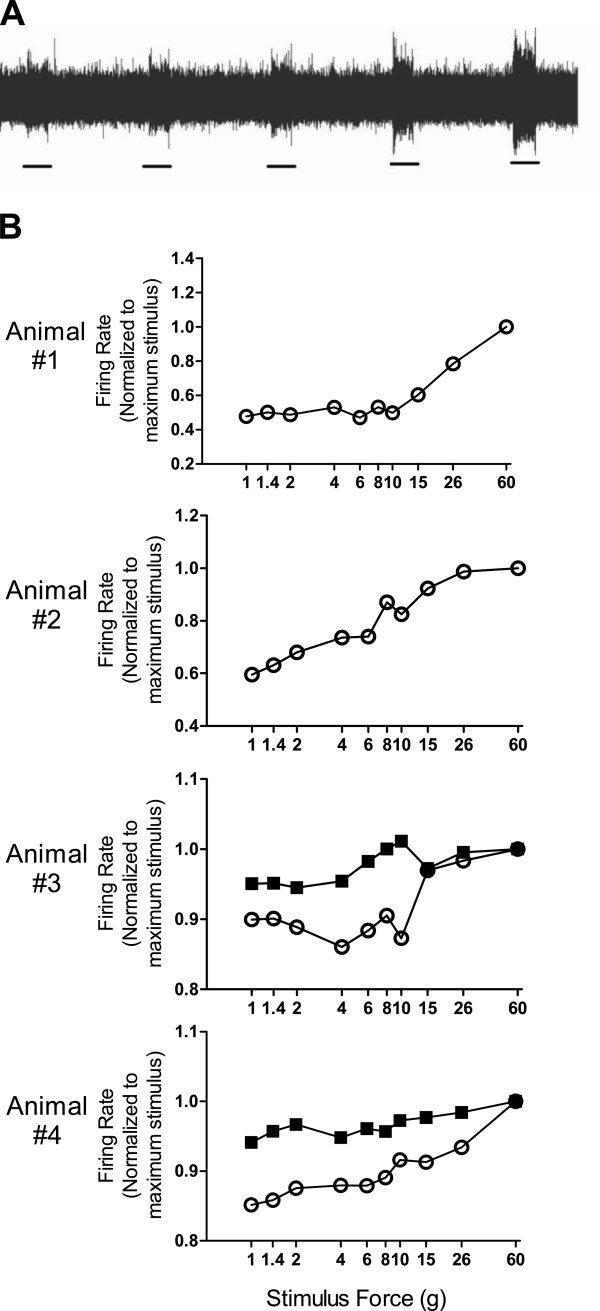
**Maxillary nerve electrical properties in monkeys.** Multiple-unit recording was performed for a nerve fiber bundle of the maxillary nerve in five monkeys with MNC. (**A**) A representative trace of multiple-unit recording. The black bars underneath the recording denote stimulation to the receptive field. The stimulus conditions were with von Frey filaments of 8, 10, 15, 26, and 60 g nominal force for 5-sec duration. (**B**) Firing rate of maxillary nerve fibers. In animals #1 and #2 only the contralateral side of the maxillary nerve was successfully recorded, while in animals #3 and #4 bilateral recording was achieved. The horizontal axis of each panel denotes the von Frey filament nominal force (in grams) that was applied to the receptive field of the maxillary nerve fibers being recorded. The maximum forge applied was 60 g. Results for each nerve fiber bundle were normalized to the data at the maximum stimulus. Open circles: firing rate data from the contralateral side of the maxillary nerve. Closed squares: firing rate data from the ipsilateral side of the maxillary nerve with MNC.

For the measurements obtained from the recorded electrophysiological data, the total number of action potentials was divided by the duration of the stimulus to derive a “firing rate” value. Since each nerve bundle had a different number of nerve fibers from other nerve bundles, the absolute values of these measurements were not directly comparable with one another. In order to provide a common basis for evaluation, values for each nerve fiber bundle were normalized to the data point at the maximum mechanical stimulus for that nerve fiber bundle. Results from the four monkeys are shown in Figure 2.

Unilateral recording of the contralateral side of maxillary nerve was obtained from animals #1 and #2. As shown in Figure 2, as the stimulation force increased, animals #1 and #2 showed increases in the firing rate. For the two animals that yielded bilateral recording data (Figure 2, animals #3 and #4), the results from contralateral side of the maxillary nerve were similar to those of animals #1 and #2, with increasing firing rate as the stimulation force increased. Interestingly, ipsilateral side of the maxillary nerve appeared “flat-lined”, with relatively little changes. Taken together, it seems that the contralateral side of the maxillary nerve displays a pattern of elevated electrical response as the stimulation force increased, whereas the ipsilateral side displayed minimum responsiveness, suggesting a possible effect of maxillary nerve compression.

## Discussion

As a way to study the consequences of damage in trigeminal nerve branches in non-human primates, we established a nerve injury model of maxillary nerve compression (MNC) in the cynomolgus macaque monkey, *Macaca fascicularis*. This is the first reported study of maxillary nerve injury model in non-human primates. Our hypothesis was that, because non-human primates show similarities to human in facial anatomy and facial nerve organization, whereas facial anatomy of rodents differs much from that of human, a maxillary nerve injury model in non-human primates will help to advance understanding of the consequences of facial nerve injury. The nerve injury from the compression appeared to be mild, as we did not observe overt changes in home-cage behavior in the monkeys.

When mechanical stimulation was applied to the facial area, monkeys with MNC displayed increased mechanical sensitivity, as the avoidance response scores were lower than those from the control animals (Figure 1B). It is noteworthy that such a change in mechanical sensitivity was evident bilaterally, even though the MNC surgery was performed unilaterally. Noteworthy is the fact that the ipsilateral side showed a more robust change, with scores significantly different from both the control and the contralateral side (Figure 1B). In rodent models of damages to trigeminal nerve branches, it has been reported that a decrease in mechanical threshold was observed bilaterally, with the ipsilateral side showing stronger sensitization than the contralateral side in rats receiving chronic constriction injury to the infraorbital nerve [1,6-8]. Also, similar results were obtained by photochemical reaction-induced partial ischemic injury to the infraorbital nerve in rats [9]. Our results in monkeys are consistent with these reports in rodents, and suggest that unilateral damage to the maxillary nerve caused bilateral changes in mechanical sensitivity, with the ipsilateral side displaying a more robust change compared to the contralateral side.

Another potential contributor to the altered somatic sensation could be from the surgical wound and accompanying inflammation. We did not observe visible inflammation, and the animals appeared not to be bothered by the surgical wound, as they did not display excessive scratching or touching of the surgical site on the face while going about with their daily activities. Also, at the time of behavioral testing, the surgical incision appeared to about to heal. Further, the somatic sensation was tested by gently brushing the areas above the lips, not at the surgical incision site over infraorbital foramen (see diagram in Figure 1A). Thus, it appears unlikely that surgical wound played a major part in altering the somatic sensation.

We also observed an effect of MNC injury on the maxillary nerve’s electrophysiological properties. Multiple-unit recording of nerve fiber bundles of the maxillary nerve was obtained from the contralateral side of two MNC monkeys, and from both sides of two MNC monkeys. As shown in Figure 2, a general trend of elevated electrical response to escalating force in mechanical stimulation was observed for the contralateral side of the maxillary nerve, with four of the five animals displaying this pattern. This suggests that the contralateral maxillary nerve in MNC model retained its electrophysiological responsiveness to mechanical stimulation applied to the receptive field. On the other hand, the maxillary nerve on the ipsilateral side displayed minimum responsiveness, as the firing rate showed rather flat response profiles (Figure 2). This indicates that after MNC injury, the ipsilateral maxillary nerve become non-responsive to mechanical stimulation applied to its receptive field.

For the ipsilateral maxillary nerve, the lack of responsiveness at the electrophysiological level is likely the consequence of MNC injury. There are two possible explanations. One is that the nerve was sensitized by the MNC injury, so that even a slight mechanical stimulation with a low force von Frey filament would cause maximum firing, therefore there was no room for further increase in electrophysiological response when the stimulation intensity escalated. Alternatively, the maxillary nerve was desensitized after the MNC injury, so that it became non-responsive to stimulation. Based on the electrophysiological data alone, it is difficult to distinguish between these mutually exclusive explanations, because the absolute values of the electrophysiological data are not directly comparable with one another, due to the fact that each nerve bundle in an electrophysiological recording had a different number of nerve fibers. However, behavioral response to mechanical stimulation offered certain clues. The ipsilateral side of the MNC surgery was more sensitive to brushing test (Figure 1B), indicating that the MNC surgery side of the face was responsive at behavioral level. This suggests that it is unlikely that the MNC side of the maxillary nerve was desensitized. It seems more likely that MNC injury resulted in a state of sensitization of the nerve at the electrophysiological level, possibly with a much reduced threshold for maximum electrical response, such that even a low intensity of stimulation to the receptive field elicited near-maximum responses of the nerve, thus giving a rather flat response profile in relation to stimulation intensity (Figure 2).

In future studies, it would be informative to examine the development of electrophysiological changes with time, with a large number of study subjects, so at different time points several monkeys could be used for electrophysiological recordings, and a time course of electrophysiological changes could be documented.

It should be noted that, compared with studies in rodent models, the monkeys used in this study were relatively heterogeneous with regard to their breeding lineage, as they were not from an established breeding colony; rather, they were bred at the laboratory animal supplier’s facility from parental monkeys captured in the wild. As such, individual variability is expected, as demonstrated by the electrophysiological response profile of monkey #2, which was different from the general pattern observed in the other monkeys (Figure 2).

## Conclusions

We carried out an initial study of a maxillary nerve compression model in non-human primates *Macaca fascicularis*. MNC did not cause overt changes in home-cage behaviors. The ipsilateral maxillary nerve appeared to show signs of increased sensitivity, as behavioral avoidance was evident, and the electrophysiological response profile of the ipsilateral nerve was different from that of the contralateral nerve. This model may serve as a non-human primate model to evaluate the effect of injury to trigeminal nerve branches.

## Methods

### Animal

Male monkeys (*Macaca fascicularis*) at the age of 3 years were used in the study. All animals were supplied by, and experiments were performed in, the laboratories at Hainan Jingang Laboratory Animal Co. Ltd., an AAALAC-accredited facility located in Hainan Province in southern China. The local climate in Hainan Province is similar to that of the natural habitat of cynomolgus monkeys. All experiment protocols were approved by the Institutional Animal Care and Use Committee (IACUC) at Hainan Jingang Laboratory Animal Co. Ltd., adhered to the guidelines of the Committee for Research and Ethical Issues of IASP, and were consistent with the National Institutes of Health Guide for the Care and Use of Laboratory Animals. Before experiments, monkeys were transferred from group housing to individual cages (80x60x70 cm, LxWxH). There were two rows of cages facing each other in the laboratory, with solid dividers between neighboring cages; thus, the monkeys could not see their immediate neighbors, but had visual contact with the monkeys in the opposite row in the room. Animals also could reach out of the cage and had limb contact with the neighboring monkeys. The room was well ventilated. Room lighting was supplied mainly by natural lighting, with some fluorescent lighting to facilitate night-time video recording of behavior. Monkeys were served three meals a day with regular monkey chow, plus seasonal fruits in the afternoon. Drinking water was provided *ad libitum*. Meal service, room cleaning, and animal care were performed by the technicians at Hainan Jingang Laboratory Animal Co. Ltd., with the supervision of certified veterinarians.

### Maxillary nerve compression (MNC) model

The monkey cages were designed with a back plate that could be pulled toward the front of the cage, so that the monkey could be restrained and immobilized. Monkeys were first sedated with 5 mg/kg i.m. ketamine in the cage, and were subsequently anesthetized with i.p. injection of 2% pentobarbital sodium at the dosage of 30 mg/kg. Additional pentobarbital sodium was given at 10 mg/kg dosage when needed. The level of anesthesia was monitored by the reflex of hind limb to mechanical pinch.

Using aseptic technique, a vertical incision was made on the right side of the face over the foreman in the skull where the maxillary nerve penetrates the bone to the outer surface of the skull (see diagram in Figure 1A). Superficial tissue and muscle were parted to expose the maxillary nerve. An L-shaped stainless steel bar about 3 mm in length made from a sterile 17-gauge injection needle was inserted into the infraorbital foreman, so that the maxillary nerve and the steel bar were next to each other, to provide moderate compression of the maxillary nerve. The incision in the skin and muscle was closed with 4.0 silk suture. Penicillin G (200,000 U) was administered after the surgery.

After the surgery, the monkey was returned to its home cage. A cloth blanket was wrapped around the monkey, and a heating fan was used to supply additional heating to help the monkey to recover from anesthesia. Visual observation was maintained until the monkey was awake and could move about in the cage.

### Behavior response to mechanical stimulation

Four days after MNC surgery, monkeys were taken out of the cage for behavioral response testing. An animal was held with its arms at the back and its legs suspended. Gentle brushing was applied with a soft hair tooth-brush, to the side of the face adjacent to the upper lip area of the monkey, where maxillary nerve innervates. A positive avoidance response was recorded when the monkey turned its head away from the side of brushing. In each test, a maximum of six successive brushings were applied to each side. The number of brushings needed to evoke an avoidance response was recorded as the avoidance response score for that side of the face. If the monkey did not respond after six brushings, the number “6” was given as the score.

Each monkey received 8 rounds of testing on each side with 5 min of inter-trial intervals between tests. The order of left vs. right side was randomized. The eight avoidance response scores were averaged as the avoidance response score for the side of the face. After testing, the monkey was returned to its home cage.

### Electrophysiology recording of maxillary nerve electric activities

Pentobarbital anesthesia was as described above. Heart beat and breathing rate were monitored. A heating pad was placed underneath the body of the animal. The body temperature was continuously monitored with an anal temperature probe, and was kept at physiological range with adjustment of the heating pad. Maxillary nerves on both sides were surgically exposed. On each side, the skin was attached to a stainless steel ring; the space of a pool was formed and was filled with pre-warmed mineral oil to keep the nerve from dehydration.

The electric activities of multiple units were recorded from a nerve bundle separated from the maxillary nerve. Nerve activities of both sides were monitored with an oscilloscope, and were recorded with a MODEL 1700 AC amplifier (A-M SYSTEMS) with different channels feeding into a DVD recorder (Sony DVD) via left or right sound channel. The signals in the amplifier were filtered with a low frequency cut-off at 300 Hz, and a high frequency cut-off at 5 KHz.

To determine the receptive field of the nerve fibers being recorded, a fine-tip soft-hair brush was used to brush confined spots in the upper lip and adjacent area. The spot that produced the strongest electric signal as shown on the oscilloscope was marked as the receptive field of the nerve fibers under recording. Thereafter, spontaneous activity of the nerve fibers was recorded for 10 min before mechanical stimulation was applied.

Mechanical stimuli were applied to the receptive field using von Frey filaments. Different stimulus intensities were applied to the receptive field in successive steps from 1g to 60g. Each stimulus lasted for approximately 5 sec, and the interval between two stimuli was 30 sec. Euthanasia was performed after the completion of the electrophysiology recording.

All recorded data were converted and transferred to computer via Powerlab 4/25 (AD Instruments), with a sampling rate for transferring data to computer at 10 KHz. The data were analyzed with Chart 5 for Windows (AD Instruments) using a plug-in software module named “spike histogram”. To determine the noise threshold, 3 separate stretches during the spontaneous activity recording without observable action potentials were selected. The highest basal potential in each piece was identified. The voltages of the 3 highest basal potentials were averaged, and the noise threshold was set as 120% of the average. The total number of action potentials during each stimulation period was acquired and was divided by the duration of the stimulus, and the result was designated as “firing rate”.

### Statistics

Facial brushing test data are expressed as mean ± SEM. Statistical significance was determined by unpaired *t* test with Welch's correction for avoidance responses (MNC ipsilateral vs. control, MNC contralateral vs. control, MNC ipsilateral vs. MNC contralateral), and by one sample *t* test for avoidance ratio.

## Abbreviations

MNC: Maxillary nerve compression; CCI: Chronic constriction injury.

## Competing interests

The authors declare that they have no competing interests.

## Authors’ contributions

NG contributed to the experimental design, data analysis, and manuscript preparation, and also performed behavioral studies. XG performed surgical procedures, and contributed to electrophysiological recording and data analysis. JZ contributed to electrophysiological recording and data analysis. GZ and MJ contributed to the experimental design and manuscript preparation. HZ, YZ, ZZ, GJJ and LY contributed to the experimental design, data analysis, and manuscript preparation. All authors read and approved the final manuscript.

## Authors’ information

Hong Zou, Yuqiu Zhang, Zhiqi Zhao, Gang Jason Jin and Lei Yu Co-corresponding authors.
